# Functional diversity and spatial association analyses at different spatial scales reveal no changes in community assembly processes along an aridity gradient in the Atacama Desert

**DOI:** 10.1038/s41598-023-47187-5

**Published:** 2023-11-14

**Authors:** Danny E. Carvajal, Andrea P. Loayza, Francisco A. Squeo

**Affiliations:** 1 Departamento de Biología, Universidad de La Serena, Casilla 554, La Serena, Chile; 2 Instituto de Ecología y Biodiversidad (IEB), Santiago, Chile; 3 Instituto Multidisciplinario de Investigación y Postgrado, Universidad de La Serena, 1720256 La Serena, Chile; 4 Centro de Estudios Avanzados en Zonas Áridas (CEAZA), La Serena, Chile

**Keywords:** Community ecology, Ecophysiology

## Abstract

The structuring of plant assemblages along environmental gradients is typically explained by shifts from competition (limiting similarity) to environmental filtering as the environment becomes more stressful. However, facilitation, weaker-competitor exclusion, environmental heterogeneity, and the colonization-competition tradeoff can also structure plant assemblages along gradients. These assembly processes act on different plant traits and organs, and their prevalence varies with respect to the spatial scale. Using patterns of functional diversity, coupled with patterns of species association at two spatial scales, here we discern the assembly processes that structure shrub communities in four localities along an aridity gradient of the Atacama Desert. At each site, we calculated functional dispersion indexes for above- and below-ground traits, and patterns of species association at a patch and neighborhood scale. Our results revealed that at the patch scale in intermediate levels of aridity, the dominant assembly process was within-site environmental heterogeneity. At the neighborhood scale, communities are assembled mainly through random processes. Nonetheless, in some communities, the dominant assembly process was competition via limiting similarity or exclusion of the weaker competitor, and these did not change along the gradient. Together, these results reveal that environmental heterogeneity and competition are the main drivers of plant community assembly in a hyper-arid environment.

## Introduction

Unravelling the processes that influence plant assemblages along environmental gradients is key to understanding patterns of plant diversity along such gradients^1^. Accordingly, the stress-dominance hypothesis (SDH)^2,3^ proposes that competition is the primary force structuring plant assemblages in the less stressful end of a gradient, but as environmental stress increases, so does the relative importance of abiotic filters. This shift is expected to lead to a change from a pattern of trait divergence to one of trait convergence^2,4^. Although some studies support the predictions of the SDH^2^, others do not^3,5–8^. The lack of consistent support to the SDH is partly explained because (1) other processes, such as facilitation, exclusion of the weaker competitor, and microenvironmental heterogeneity, can also structure plant assemblages along environmental gradients^7,9–13^, and (2) because the prevalence of these processes is scale-dependent; that is, the importance of the different assembly processes changes across different scales^4,14,15^.

Patterns of trait convergence and divergence change along environmental gradients; however, a simple trend of increasing or decreasing functional divergence is not an obvious indicator of the underlying assembly processes that structure plant assemblages^11^. For example, the widespread expectation of the SDH is that trait convergence should occur in the most stressful abiotic conditions, whereas trait divergence should be more prevalent in less stressful environments^2,3^; however, competition can also lead to trait convergence via weaker-competitor exclusion or hierarchical competition^13,16^. Alternatively, the stress gradient hypothesis (SGH) predicts that facilitation and competition vary inversely along environmental gradients, with facilitation becoming more common with increasing abiotic stress because it ameliorates micro-environmental conditions for less stress-tolerant species^9^. Consequently, facilitation can increase the functional diversity of plant assemblages, which should result in high trait divergence^6,7,17–19^. Furthermore, some studies suggest that the effect of facilitation can decrease when the environment becomes highly stressful^20,21^, leading to competition being the dominant driver of plant assemblages at both ends of the gradient^22^. Therefore, to better understand the processes that shape plant communities, patterns of functional diversity should be combined with patterns of spatial association^11,23^.

In the context of non-random patterns of species association^24^, segregation is typically associated with competition, while aggregation is associated with facilitation^24,25^. However, various factors can contribute to these spatial patterns^10,11,14,23^. For example, species may segregate due to either competition or environmental heterogeneity, where different species with varying abiotic tolerances can occupy distinct microenvironments within a community^11,12^. Conversely, aggregation may result from facilitation, seed trapping, dispersal limitation, and environmental heterogeneity, where plant species tend to cluster together in favorable habitats^10,14^. Segregation can also arise from differences in species dispersal abilities, resulting in a trade-off between their colonization and competitive abilities^26–29^.

Several assembly processes can result in trait convergence or divergence and species aggregation or segregation along an environmental gradient. However, the relative importance of these processes varies depending on the spatial scale under consideration^4,14,15^. Accordingly, competition and facilitation can change along environmental gradients at small (i.e., neighborhood) spatial scales^11–13,23^, while abiotic filtering tends to drive trait convergence at larger (i.e., patch) spatial scales. Conversely, environmental heterogeneity can lead to either trait convergence or divergence^4,10,23,30^. Additionally, at larger spatial scales, differences in the dispersal and competitive abilities of plants can also shape plant assemblages (via a colonization-competition trade-off) and drive patterns of trait divergence^26–29^. Therefore, it is crucial to examine the assembly processes operating at different spatial scales to untangle the factors that shape plant assemblages along environmental gradients.

A further complexity in understanding how plant assemblages are organized along environmental gradients is that different assembly processes can operate within a single locality but on different traits or plant organs^2,3,5–8^. Consequently, if all plant traits are examined jointly, a dominant assembly process in a locality may not emerge. For example, Spasojevic and Suding^6^ found that when leaf traits and plant height were analyzed jointly, the pattern of trait dispersion of subalpine grassland communities along an environmental gradient did not change. However, when they examined each trait separately, divergence in plant height and leaf area increased at both ends of the gradient, suggesting that competition and facilitation, determined the structuring of these grassland assemblages in the least and most stressful ends. Similarly, in semi-arid scrubland communities along an aridity gradient, Gross et al.^7^ found that the signal of plant height shifted from competition in the less arid end of the gradient to facilitation towards the more arid end; conversely, the intensity of competition increased as the environment became more arid for the specific leaf area (SLA). These and other studies have focused on above-ground traits based on the assumption that aboveground competition is high due to light limitation (e.g., Chalmandrier et al.^15^; de Belo et al.^23^); however, when soil resources (e.g., water availability and nutrients) are limiting, below-ground traits play critical roles in response to environmental filtering (e.g., León et al.^31^) and concerning competitive and facilitative interactions^10,32–36^. For example, Butterfield, et al.^35^ studying perennial grassland communities along an aridity gradient in Arizona found that as aridity increased, variation in specific root length (SRL) also increased, promoting species coexistence via niche partitioning of soil resources. Consequently, incorporating below-ground traits into assembly studies can improve our understanding of the processes that structure plant assemblages, particularly in arid ecosystems.

The Atacama Desert provides an ideal model for studying how different assembly processes structure shrub communities due to its steep aridity gradient^36^. Additionally, it has been proposed that the main processes structuring shrub assemblages at the neighborhood scale along this gradient shift from competition to facilitation (mediated by below-ground traits) as the environment becomes increasingly arid^10^. Furthermore, evidence indicates that along the aridity gradient, shifts in plant resource use strategies of the above-ground traits are influenced by aridity. In contrast, those mediated by below-ground traits respond to aridity and biotic interactions^36^. In light of this information, two expectations emerge concerning the neighborhood scale: (1) below-ground traits should align with the predictions of the SGH^9^, and (2) above-ground traits should align with the SDH^2,3^. On the other hand, localities along this gradient exhibit high soil resource heterogeneity, especially at intermediate levels of aridity. Consequently, at the patch scale, the effect of soil heterogeneity should be greater at intermediate levels of aridity than at either end of the gradient.

**Figure 1:**
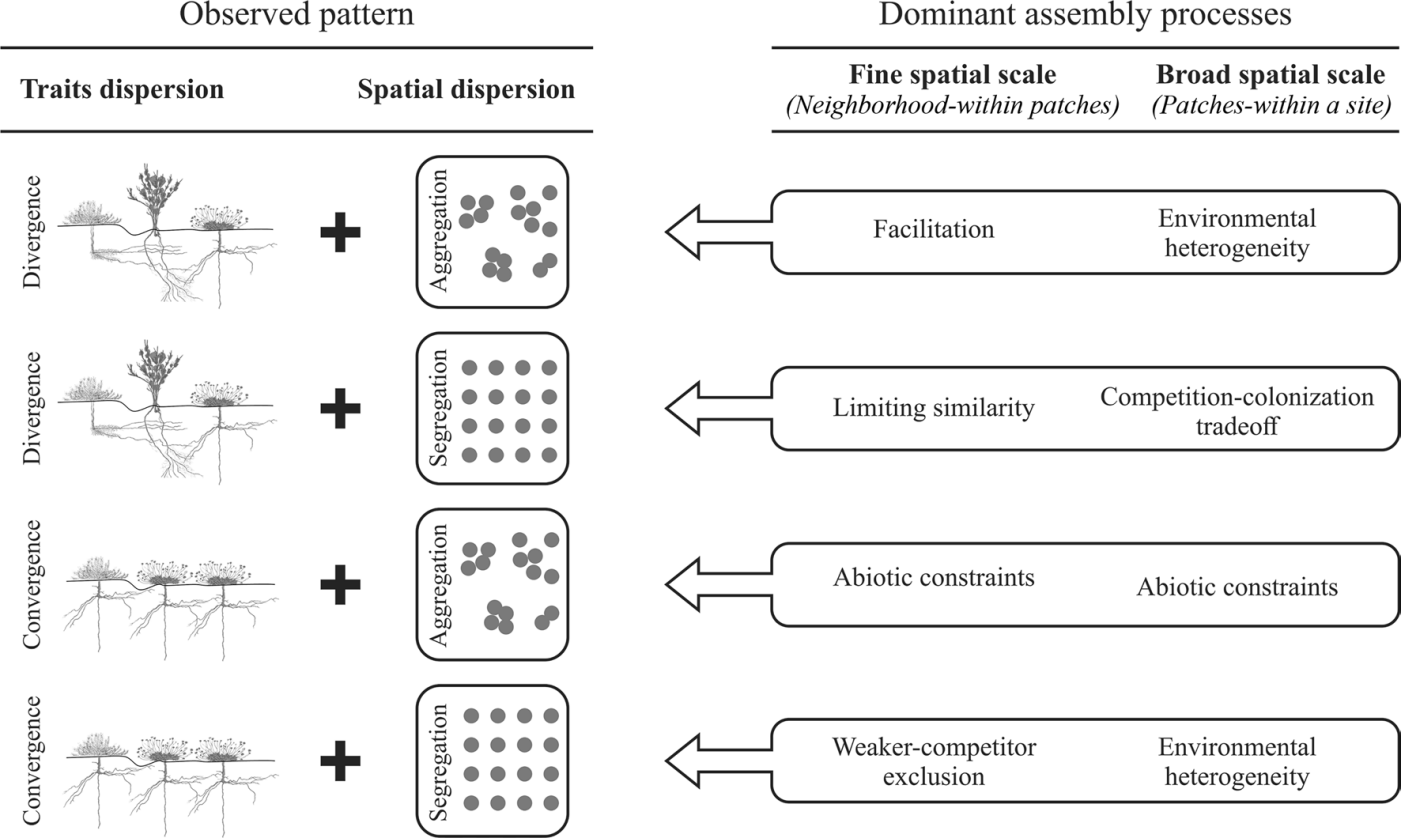
Conceptual diagram illustrating the predictions derived from patterns of spatial association and functional diversity at different scales. At finer spatial scales (neighborhood), the combination of spatial association patterns and functional diversity provides four possible scenarios regarding the dominant assembly processes: (1) Facilitation is inferred when species are spatially aggregated and traits exhibit divergence; (2) limiting similarity is expected to prevail when species are spatially segregated and traits show a pattern of divergence; (3) abiotic constraints are assumed to be at play when species are spatially aggregated and traits exhibit convergence and; (4) weaker-competitor exclusion is inferred if species are spatially segregated, and traits show convergence. At a broader scale (among patches), we anticipate the following scenarios: (1) Habitat filtering is expected to result in trait convergence and spatial aggregation (abiotic constraint), (2) micro-Environmental Filtering (Environmental Heterogeneity) is indicated by trait convergence and spatial segregation, because species with varying ecological tolerances or resource-use strategies occupy different habitats or patches within a site. The effects of environmental heterogeneity can also be detected if plants are spatially aggerated and their traits diverge because plant species are occupying the more favorable patches within a site (environmental heterogeneity). Finally, (3) a competition-colonization tradeoff will lead to trait divergence and spatial segregation of species.

In this study, we employ a trait-based approach in conjunction with analyses of spatial patterns of species aggregation at two spatial scales (neighborhood and patch scale; Fig. 1) to examine if the assembly processes shaping shrub communities in the Atacama Desert change along the aridity gradient and across spatial scales. Specifically, we test two hypotheses. First, if the environmental heterogeneity at the patch scale changes along the gradient. We predict that shrub communities in sites with higher soil resource heterogeneity will exhibit either trait divergence and spatial aggregation or trait convergence and spatial segregation (Fig. 1). Second, we test if, at the neighborhood scale, increasing aridity leads to below-ground traits shifting from competition to facilitation (in accordance to the SGH), and to above-ground traits shifting from competition to habitat filtering (in accordance to the SDH). We predict that with increasing aridity, there will be a clinal change in below-ground traits, from segregation to aggregation, coupled with a pattern of trait divergence regardless of the level of aridity (Fig. 1). For above-ground traits; however, we predict a clinal change from segregation and divergence to aggregation and convergence (Fig. 1). At this scale, pooling of all traits should result in a shift from a pattern of trait divergence to a random pattern with increasing aridity. This is because the patterns of divergence and convergence for above- and below-ground traits, should cancel each other in the more arid end of the gradient.

## Results

**Figure 2:**
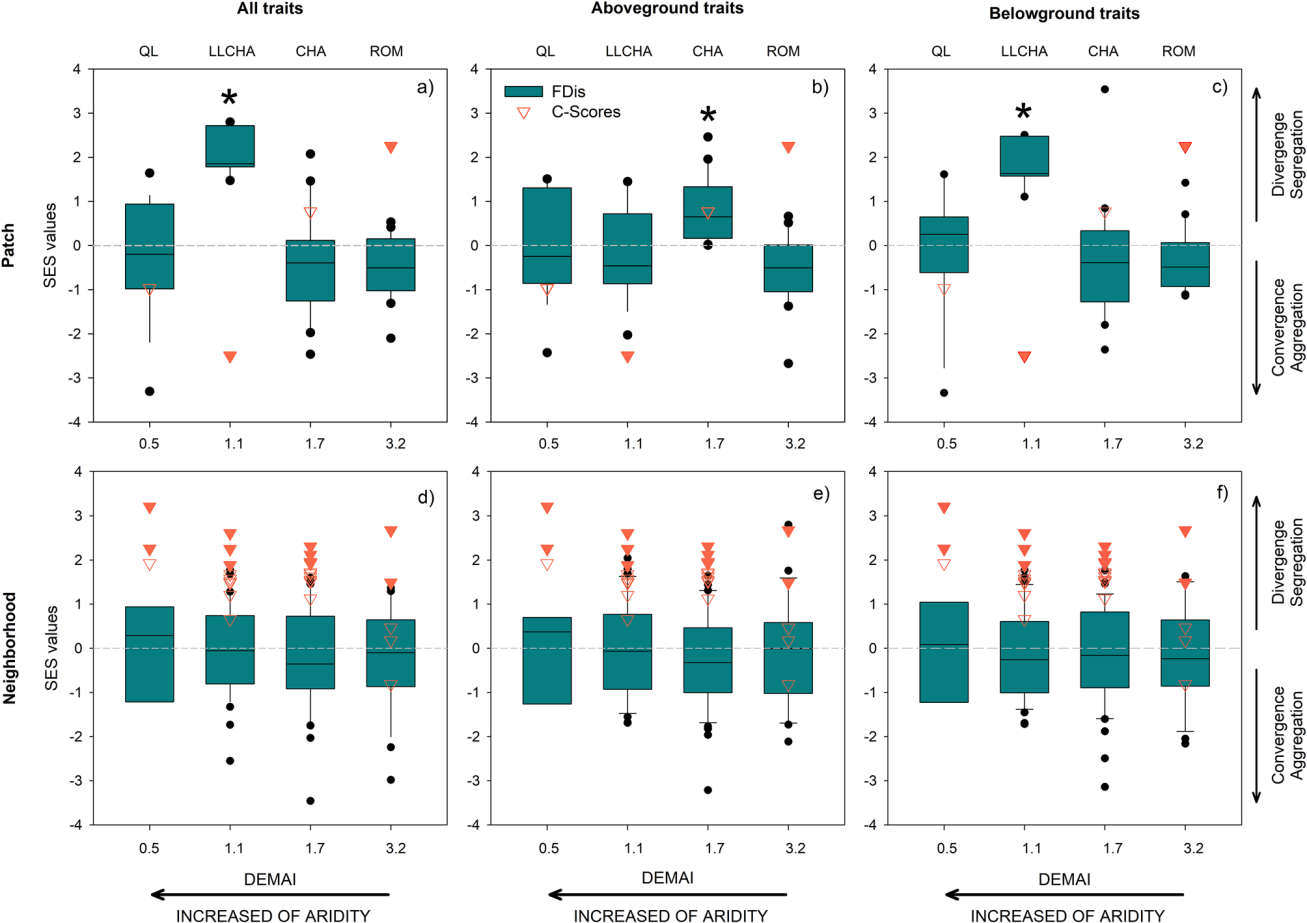
Mean standardized effect size (SES) of the functional diversity index (FDis) and SES of C-Scores (triangles) along the aridity gradient in the Atacama Desert as indicated by DEMAI (De Martonne Aridity Index) at the patch and neighborhood scale for: (**a,d**) all traits, (**b,e**) aboveground traits, (**c,f**) belowground traits. *Indicates significant statistical differences from the null expectation based on two tailed t-tests for FDis and, filled orange triangles represent SES C-scores values significantly different from the null expectation. Lower DEMAI index values indicate greater aridity. *QL* Quebrada El León, *LLCHA* Norte Llanos de Challe, *CHA* Chañaral de Aceituno, *ROM* Romeral.

The mean standardized effect size (SES) of the functional dispersion index (FDis) was only significantly divergent at the patch scale and not at the neighborhood scale (Fig. 2). Specifically, the SES of FDis at the patch scale was significantly divergent in the two sites with intermediate levels of aridity (LLACHA and CHA). This was true when analyzing all traits together, as well as separately for below-ground traits at LLCHA (Fig. 2a,c) and above-ground traits at CHA (Fig. 2b). C-Scores revealed that shrub species were aggregated only in LLCHA, the second most arid location (Fig. 2a–c). In contrast, they were segregated in the wettest site (ROM; Fig. 2a–c) and were not different from random in the other sites (CHA and QL; Fig. 2a–c). At the neighborhood scale, the FDis did not differ significantly from the null distribution along the aridity gradient when all traits were examined jointly nor when above- and below-ground traits were examined separately (Fig. 2d–f). Nevertheless, some plots in all sites showed significant differences from the null distribution, indicating divergence or convergence (Fig. S1d–f). Additionally, at the neighborhood scale and irrespective of the aridity level, the SES of C-scores that significantly deviated from the null distribution indicated species segregation (Fig. 2d–f, Fig. S1d–f).

## Discussion

Our findings suggest that, at the patch scale, in the site with intermediate aridity levels (LLCHA), environmental heterogeneity was the main driver in structuring shrub assemblages; no distinct assembly process was evident in the other localities (Fig. 1a–c). These results provided partial support for our initial hypothesis, indicating that environmental heterogeneity exerts a more pronounced influence in locations with greater overall heterogeneity. Environmental heterogeneity in soil resources can shape plant communities by either enabling species with differing abiotic tolerances or resource utilization strategies to occupy different habitats within sites^11,12^ or by facilitating the coexistence of multiple species within more favorable patches^10,14^. In our study, the observed pattern in LLCHA, where plants were spatially aggregated and exhibited divergent traits (Fig. 2a,c), aligns with the latter explanation. Our findings also suggest that below-ground traits may have significantly contributed to the level of divergence observed when examining all plant traits collectively. In LLCHA, plants are likely using the most favorable habitats, and the reduction in niche overlap is achieved through greater trait divergence among species within a patch. This differentiation is largely influenced by below-ground traits, which may reduce interspecific niche overlap^12,13^. For example, in this same locality, Carvajal et al.^36^ observed a high level of diversity in root dry matter content (RDMC). This trait is associated with root lifespan, hence the diversity of RDMC may facilitate temporal partitioning of soil resources, thereby reducing competition among species^13^.

In CHA, plants exhibited divergent above-ground traits, but the spatial distribution of species was random. The higher functional diversity in CHA can be attributed to the greater variation in leaf traits associated with resource acquisition strategies, including leaf area, leaf dry matter content, and long-term water use efficiency^36^. However, because shrub species were randomly distributed in space, we cannot conclusively determine that the trade-off between colonization and competition is the primary mechanism structuring these shrub communities. This randomness in spatial distribution might contribute to species coexistence at large spatial scales^26–29,32^.

At the neighborhood scale, while c-scores indicated spatial segregation of shrubs, we did not observe a shift in the dominant assembly process along the aridity gradient. This remained true whether we analyzed all traits collectively or separately for above- and below-ground traits, as the FDis did not significantly differ from the null distribution across the aridity gradient (Fig. 2d–f). Consequently, we found no support to the hypothesis that as aridity increases, below-ground traits should reveal a shift from competition to facilitation (as predicted by the SGH), while above-ground traits should indicate a shift from competition to habitat filtering (as predicted by the SDH). At the neighborhood scale, it appears that shrub species exhibit a random assembly pattern across all sites along the gradient. There are several possible explanations for these results. First, it is worth noting that we did not account for intraspecific trait variation (ITV) in our analysis. Because ITV reduces the similarity among neighboring species in response to biotic interactions, it can increase the probability of detecting non-random assembly processes in plant communities (e.g., Jung et al.^37^; Siefert^38^). However, even if we had considered ITV, it is unlikely that it would have contributed to detecting assembly processes in our system. This is because among-patch ITV in our system is lower than 17% (Carvajal et al. unpublished data), and a substantial effect in detecting assembly processes typically requires ITV to exceed 30%^39^. Second, disparities in dispersal limitation can obscure the effects of the assembly process and result in random patterns. However, because dispersal limitation is believed to be more pronounced at larger spatial scales^23^, it is less likely to generate random patterns at the neighborhood scale. Third, intransitive competition (i.e., when there is no unique best competitor; *sensu* Soliveres et al.^40^) could also lead to a random pattern. This occurs because intransitive competition produces a spectrum of trait values, that may not necessarily result in trait convergence or divergence^41^. Fourth, neutrality and ecological drift can contribute to random trait patterns. This happens because (a) individual fitness becomes independent of species identity and community composition (i.e., there is functional equivalence) and (b) the stochastic natures of births and deaths can lead to poor regulation of plant abundance^42,43^. Fifth, a random pattern can also emerge when multiple assembly processes simultaneously act on plant assemblages, concealing the underlying processes that underlie divergence or convergence^30,44^. In the Atacama Desert, at least three of these factors appear to be contributing to the random assembly of shrub communities: intransitive competition, neutrality and ecological drift, and multiple assembly processes acting simultaneously. Hence, further studies are required to untangle the complex processes influencing plant communities in this desert. However, despite these explanations, it is noteworthy that a few plots deviated significantly from the null distribution at this scale, both in terms of the SES of C-Scores and the SES of the FDis. These deviations suggest that regardless of the aridity level in the Atacama Desert, plant competition may play a central role in shaping plant assemblages, either through limiting similarity or by excluding the weaker competitors. This observation holds true not only for above- and below-ground traits but also when considering all traits together. These findings contrast theoretical and empirical studies that show a shift from competition to facilitation (e.g., Bertness and Callaway^9^; López et al.^10^) or a shift from competition to environmental filtering (e.g., Coyle et al.^3^) towards the most stressful end of the gradient.

In conclusion, our study revealed that at the patch scale, environmental heterogeneity emerged as the dominant assembly processes in only one site. At neighborhood scale, we observed a weak signal of competition as the dominant assembly process. Specifically, signs of competition were present in all sites in all sites but were only evident in a few plots. Our results underscore the need to apply both trait-based metrics and spatial association analyses at different spatial scales to understand community assembly. For example, at the neighborhood scale, divergence detected by the functional dispersion index could indicate either competition or facilitation, while convergence might stem from abiotic constraints or weaker-competitor exclusion. Conversely, segregation patterns identified by C-scores suggest competition, however they do not clarify whether it is due to limiting similarity or weaker-competitor exclusion. Employing both methods allows us to infer not only the predominant process shaping plant communities but also the specific drivers behind these processes.

## Methods

### Study site

We conducted this study in the Atacama Desert (Chile) along an aridity gradient that expands approximately 440 km (from 26° 57ʹ S to 29° 43ʹ S). Rainfall across this area is limited to the winter months^45^, and is more variable towards the north^46^. The mean annual precipitation along the gradient ranges between 14 and 80 mm, while the mean annual temperature is relatively constant, fluctuating between 15 and 17 °C (Table 1). We selected four sites along the gradient that differ in their degree of aridity, which was estimated using De Martonne’s aridity index: DEMAI = MAP/(MAT + 10)^47^, where MAP and MAT are the mean annual precipitation and temperature, respectively. From the most to the least arid site, these are: Quebrada El León (QL), Norte de Llanos de Challe (LLCHA), Chañaral de Aceituno (CHA) and Romeral (ROM) (Table 1). Of these, LLCHA exhibited the highest variability in soil physico-chemical properties (Table 1).

**Table 1:** Location and climatic characteristics of the four study sites.

Locality	Coordinates	Climatic characteristics			Variability in within site soil properties—CV (%)	
		MAP (mm)	MAT (°C)	DEMAI	Soil chemistry	Soil physics
QL	26° 57ʹ 53ʹʹ S–70° 45 59ʹʹ W	14	17.2	0.5	0.54	0.36
LLCHA	27° 59ʹ 21ʹʹ S–71° 07ʹ 22ʹʹ W	28	16.6	1.1	0.72	0.54
CHA	29° 06ʹ 16ʹʹ S–71° 27ʹ 18ʹʹ W	45	16.1	1.7	0.62	0.18
ROM	29° 43ʹ 48ʹʹ S–71° 19ʹ 24ʹʹ W	80	15.3	3.2	0.38	0.44

The De Martonne aridity index (DEMAI) was calculated as MAP/ (MAT + 10); thus, lower values reveal greater aridity. Climate data (Mean annual precipitation, MAP; Mean annual temperature, MAT) were obtained from Dirección General de Aguas, Chile (www.dga.cl) and from CEAZA-Met weather station (www.ceazamet.cl). Values of coefficients of variation (CV) in within site soil properties were calculated as (Standard deviation/mean) × 100.

*QL* Quebrada El León, *LLCHA* Norte Llanos de challe, *CHA* Chañaral de Aceituno, *ROM* Romeral.

### Data collection

At each site, vegetation was sampled in stabilized dunes (sandy soils) of west-facing, gentle slopes (< 5%)^36^. The sampling procedure was carried out at two scales: the patch scale (100 m^2^ plots) and the neighborhood scale (5 m^2^ plots). For the patch scale, we established 20 plots of 50 × 2 m separated by at least 100 m in each of the four sites (N = 20/site, except for QL that had 22 plots). We recorded all plant species present in each plot, as well as the number of individuals per species, which was used to estimate the relative abundance of species per plot. For the neighborhood scale, we selected 10 of the 20 plots established per site (except for QL that hat 11 plots), divided the area of each selected plot into five subplots and recorded the number of individuals per species in three 1 m^2^ quadrants within each subplot.

### Trait measurements

We measured plant height and a set of leaf, stem and root traits following standardized protocols^48^. At each site, we randomly selected 50 individuals to measure all leaf traits, except leaf chemistry traits for which we sampled five individuals per species per site. Similarly, only five individuals per species per site were used to measure stem traits. We selected only three individuals per species per site to measure coarse-root traits and five individuals to measure fine-root traits because the methodology used to quantify roots traits is destructive^36^. In total, we measured 14 traits: Plant height (cm), leaf area (LA—cm^2^), specific leaf area (SLA; the ratio of leaf area to leaf mass (cm g^−2^)), leaf dry matter content (LDMC; the ratio of leaf dry mass to fresh mass (mg g^−1^)), leaf nitrogen concentration (LNC (%)), foliar carbon isotope ratio (δ^13^C (‰)), carbon to nitrogen ratio (CN_leaf_ (mg·mg^−1^)), stem wood density (WDs; the ratio of oven dry mass to green volume (g·cm^-3^)), root dry matter content (RDMC; the ratio of root dry mass to fresh mass (mg·g^−1^)), specific root length (SRL; the ratio of root dry mass to length (g·cm^−1^)), root nitrogen concentration (RNC (%)), carbon to nitrogen ratio (CN_root_ (mg·mg^−1^)), root depth distribution (β index) and root wood density (WDr; the ratio of oven-dried mass to green volume (g·cm^−3^)). For a detailed description of trait measurements and their functional significance see^36^.

### Data analysis

Trait-based assembly processes were estimated using the functional dispersion index (FDis; Laliberté and Legengre^49^); this index can accommodate multiple traits, considers species abundance and is independent of species richness^49^. Calculations of the FDis at both scales involved computing the trait means for each site using the dbFd function in the FD package^50^. To test for non-random assembly processes along the aridity gradient at each spatial scale, we conducted analyses for all traits combined, as well as above- and below-ground traits separately. The species pool was restricted according to the scale^51^: at the patch scale, we used the whole set of species (i.e., all of the species found in all of the plots within a site), while at the neighborhood scale, we used all of the species found within a single plot. To calculate null distributions, we generated 9999 randomly assembled communities using constrained randomizations, which keep fixed the total number of species and abundance in the samples (i.e., plots or subplots) and reshuffle species abundances while keeping the same number of species (species richness) within samples^24,52^. Subsequently, we calculated the standardized effect size (SES) as: SES = (T_obs_ − T_null_)/SD_null_, where T_obs_ is the observed FDis, T_null_ is the mean FDis of the null distribution, and SD_null_ is the standard deviation of the null distribution. To test whether SES values of the FDis were significantly lower (SES < 0 = convergence) or greater (SES > 0 = divergence) than expected, we estimated p-values using two-tailed tests of the quantile scores, which test whether an observed FDis value falls outside the null distribution using the randomizeMatrix function in Picante package^53^. The final number of randomizations at the patch and neighborhood scale varied (Table S1) because some samples contained less than three species, which rendered it impossible to perform null models.

Patterns of interspecific spatial association were estimated using the checkerboard score (C-score; Stone and Roberts 1990). C-score calculations were performed using species occurrence data summarized into presence–absence matrices (species in rows, quadrats in columns) for each scale (patch and neighborhood). We then tested for non-random pairwise species associations by constructing null models with 10,000 randomly assembled communities for each scale using fixed-equiprobable and fixed-proportional algorithms. Specifically, we used the fixed-equiprobable algorithm at the patch scale, which keeps the total number of species in a site fixed and randomizes species occurrences among plots. Thus, this algorithm assumes that plots are homogeneous and can be occupied by any species within a site and thus, is appropriate to detect the signal of abiotic constraints. Conversely, at the neighborhood scale, we used the fixed-proportional algorithm, which also keeps the total number of species in a plot fixed but randomizes species within subplots proportional to the observed species richness within a plot. Because this algorithm maintains the differences in species occurrences among plots but randomizes occurrences within a plot, it is apt to detect the signal of biotic interactions^24^. We calculated the SES using the formula described above. To examine patterns of spatial association, we tested whether C-scores values were significantly lower (SES < 0 = aggregated) or greater (SES > 0 = segregated) than expected by comparing the observed C-score of each matrix to the 95% confidence interval of simulated C-scores generated by the null models. These analyses were performed using the cooc_null_model function in the EcoSimR package^54^. All statistical analyses were performed using the R statistical environment^55^.

### Ethics declarations

Experimental research and field studies on plants (either cultivated or wild), including the collection of plant material, must comply with relevant institutional, national, and international guidelines and legislation.

### Supplementary Information

Supplementary Information.

## Data Availability

All data generated or analyzed during this study are included in this published article (and its Supplementary Information files).
