# Supplementary material for: Functional diversity and spatial association analyses at different spatial scales reveal no changes in community assembly processes along an aridity gradient in the Atacama Desert
Source: Sci Rep. 2023 Nov 14;13:19905. doi: 10.1038/s41598-023-47187-5 (PMC10646005; doi:10.1038/s41598-023-47187-5)
Supplement: Supplementary file 1 — Supplementary Information. [file 41598_2023_47187_MOESM1_ESM.docx]

# SUPPLEMENTARY MATERIALS

Functional diversity and spatial association analyses at different spatial scales reveal no changes in community assembly processes along an aridity gradient in the Atacama Desert

Danny E. Carvajal^1,3^, Andrea P. Loayza^1,2,3,^, And Francisco A. Squeo^1,3,4,^

^1^Departamento de Biología, Universidad de La Serena, Casilla 554, La Serena, Chile

^2^Instituto Multidisciplinario de Investigación y Postgrado, Universidad de La Serena, La Serena, Chile

^3^Instituto de Ecología y Biodiversidad (IEB)

^4^Centro de Estudios Avanzados en Zonas Áridas (CEAZA), La Serena, Chile

^*^Corresponding author: Danny E. Carvajal; e-mail: dcarvajal@userena.cl

Figure S1. Individual values of the standardized effect size (SES) of the Functional Diversity index (FDis) (circles) and SES of C-Scores (triangles) with the degree of aridity in the Atacama Desert as indicated by DEMAI (De Martonne Aridity Index) at the patch and neighborhood scale for: a and d) all traits, b and e) aboveground traits, c and f) belowground traits. Filled blue circles and orange triangles represent SES values significantly different from the null expectation. Lower DEMAI index values indicate greater aridity. Site acronyms: QL = Quebrada El León, LLCHA = Norte Llanos de Challe, CHA = Chañaral de Aceituno, ROM = Romeral


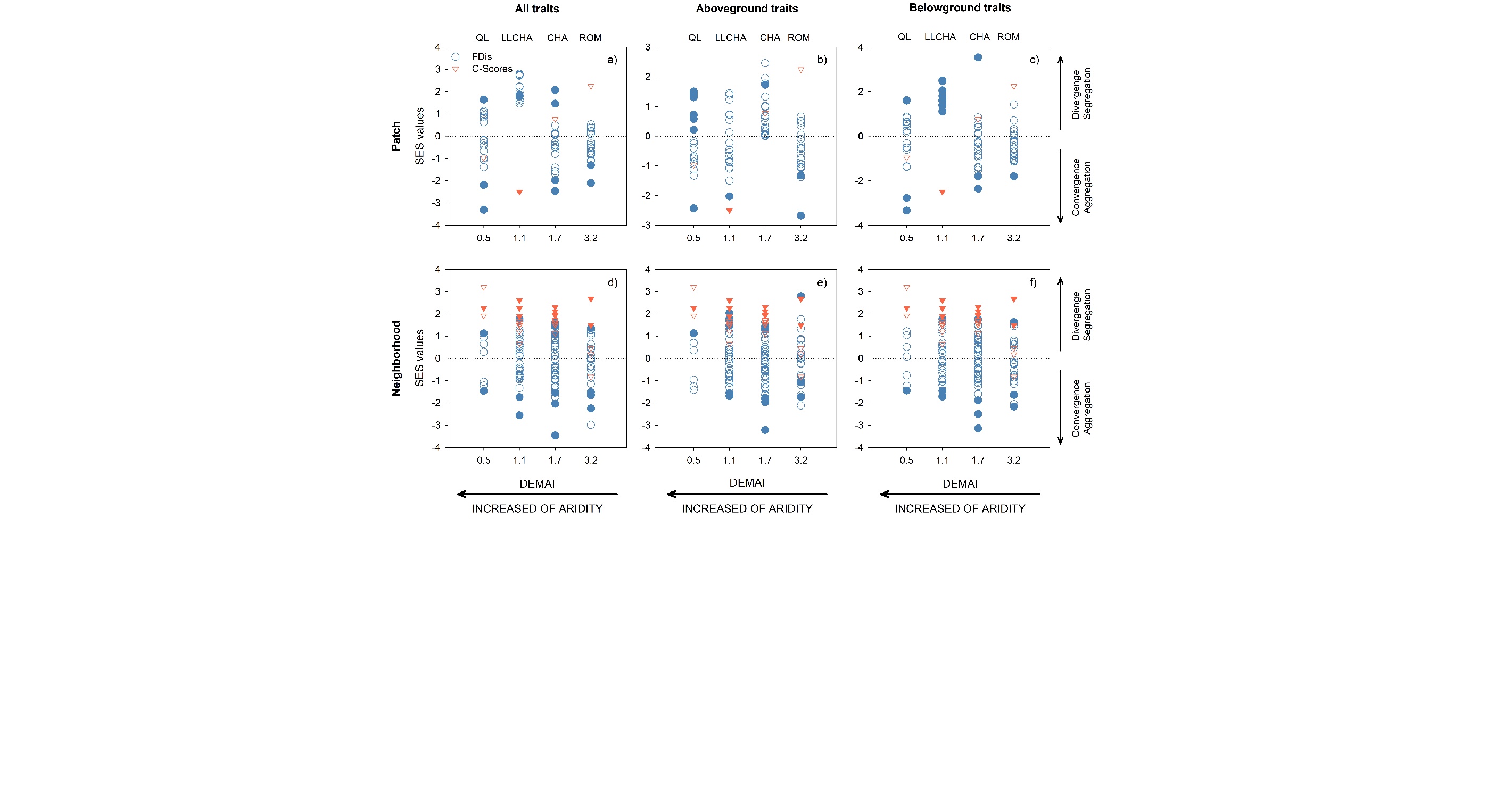


Table S1. Final number of randomizations performed at each scale at each site. Site acronyms: QL = Quebrada El León, LLCHA = Norte Llanos de Challe, CHA = Chañaral de Aceituno, ROM = Romeral

| Site | Total number of randomizations performed | |
| --- | --- | --- |
|  | Patch scale | Neighborhood scale |
| QL | 19 | 7 |
| LLCHA | 19 | 32 |
| CHA | 20 | 46 |
| ROM | 20 | 23 |

Table S2. Standardized effect size (SES values) of the checkerboard score (C-scores) and the functional dispersion index (FDis) for All traits, Aboveground traits, and Belowground traits at each plot at the patch scale. Site acronyms: QL = Quebrada El León, LLCHA = Norte Llanos de Challe, CHA = Chañaral de Aceituno, ROM = Romeral.

| Site | Scale | Plot | SES values | | | |
| --- | --- | --- | --- | --- | --- | --- |
|  |  |  | C-Scores | FDis All traits | FDis Aboveground traits | FDis Bellowground traits |
| QL | Patch | 2 | -0.96717 | 0.836165 | 0.2146948 | 1.6138689 |
| QL | Patch | 3 |  | -0.1691774 | -0.758705 | 0.1762827 |
| QL | Patch | 4 |  | -1.052124 | -0.8572278 | -1.355999 |
| QL | Patch | 6 |  | 1.1341344 | 1.3067842 | 0.8777496 |
| QL | Patch | 7 |  | -0.4256375 | -0.245732 | -0.520842 |
| QL | Patch | 8 |  | 1.6419392 | 1.4218771 | 1.5961244 |
| QL | Patch | 9 |  | 1.0135271 | 1.3802844 | 0.5482484 |
| QL | Patch | 10 |  | -0.979112 | -0.9457817 | -1.3783796 |
| QL | Patch | 11 |  | -0.4245124 | -0.1671052 | -0.3133981 |
| QL | Patch | 12 |  | -2.1907798 | -1.1271268 | -2.7718956 |
| QL | Patch | 13 |  | 0.8822235 | 0.7210261 | 0.8353913 |
| QL | Patch | 14 |  | 0.9370586 | 1.3682809 | 0.6499493 |
| QL | Patch | 15 |  | 0.6301702 | 0.5800088 | 0.4490611 |
| QL | Patch | 16 |  | -0.6527803 | -0.715952 | -0.5042562 |
| QL | Patch | 17 |  | -0.3204657 | -0.3997995 | 0.2532341 |
| QL | Patch | 18 |  | -0.1997243 | -0.6776369 | 0.6326368 |
| QL | Patch | 19 |  | -3.3048951 | -2.4281494 | -3.3392078 |
| QL | Patch | 20 |  | -1.3880838 | -1.3299391 | -0.6126233 |
| QL | Patch | 22 |  | 1.0907178 | 1.507453 | 0.5583962 |
| LLCHA | Patch | 1 | -2.4948 | 2.717512 | 1.3978388 | 2.478756 |
| LLCHA | Patch | 2 |  | 2.730119 | 0.5484339 | 2.505468 |
| LLCHA | Patch | 4 |  | 2.799279 | -0.5468199 | 2.476614 |
| LLCHA | Patch | 5 |  | 2.744171 | 1.4487555 | 2.479161 |
| LLCHA | Patch | 6 |  | 2.763843 | -0.4473382 | 2.503994 |
| LLCHA | Patch | 7 |  | 1.959239 | 0.717556 | 1.804463 |
| LLCHA | Patch | 8 |  | 2.215192 | -0.792691 | 2.05992 |
| LLCHA | Patch | 9 |  | 1.84165 | -2.025671 | 1.631442 |
| LLCHA | Patch | 10 |  | 2.224558 | -1.0935282 | 2.055615 |
| LLCHA | Patch | 11 |  | 1.799736 | 0.1316266 | 1.591105 |
| LLCHA | Patch | 12 |  | 1.644451 | -0.459463 | 1.369987 |
| LLCHA | Patch | 13 |  | 1.849321 | -1.4933145 | 1.625477 |
| LLCHA | Patch | 14 |  | 1.81828 | -1.0488814 | 1.588407 |
| LLCHA | Patch | 15 |  | 1.68728 | -0.2259684 | 1.404502 |
| LLCHA | Patch | 16 |  | 1.472186 | 1.2309072 | 1.106157 |
| LLCHA | Patch | 17 |  | 1.78235 | -0.6703995 | 1.57458 |
| LLCHA | Patch | 18 |  | 2.231797 | -0.8478911 | 2.031194 |
| LLCHA | Patch | 19 |  | 1.78692 | 0.7350476 | 1.587207 |
| LLCHA | Patch | 20 |  | 1.566486 | -0.8665194 | 1.129234 |
| CHA | Patch | 1 | 0.77144 | 2.07394399 | 0.616468931 | 3.5389693 |
| CHA | Patch | 2 |  | 0.07453912 | 0.306950555 | 0.5605851 |
| CHA | Patch | 3 |  | -0.37936296 | 0.682232408 | 0.1007489 |
| CHA | Patch | 4 |  | 0.48321253 | 0.988359246 | -0.2430328 |
| CHA | Patch | 5 |  | -0.48249512 | 0.155382335 | -0.8203986 |
| CHA | Patch | 6 |  | -1.40631321 | 1.33215989 | -1.4278292 |
| CHA | Patch | 7 |  | -2.4638293 | 2.45943464 | -2.358841 |
| CHA | Patch | 8 |  | 0.12436759 | 0.075016449 | 0.1430107 |
| CHA | Patch | 9 |  | -0.4079989 | 0.49381944 | -0.3069346 |
| CHA | Patch | 10 |  | -1.68081763 | 1.774138123 | -1.3853015 |
| CHA | Patch | 11 |  | 0.09349762 | 0.187522892 | 0.4219114 |
| CHA | Patch | 12 |  | 0.15278374 | 0.054546078 | 0.3983408 |
| CHA | Patch | 13 |  | -0.53271821 | 0.689304839 | -0.2822152 |
| CHA | Patch | 14 |  | 1.46309836 | 1.735960524 | 0.8420175 |
| CHA | Patch | 15 |  | -0.79664095 | 1.017024253 | -0.4707251 |
| CHA | Patch | 16 |  | -1.97372845 | 1.956551124 | -1.522129 |
| CHA | Patch | 17 |  | -0.41519684 | 0.025126734 | -0.94616 |
| CHA | Patch | 18 |  | -0.28508554 | 0.005723638 | -0.6744412 |
| CHA | Patch | 19 |  | -1.56975685 | 1.329241584 | -1.7997237 |
| CHA | Patch | 20 |  | -0.26966446 | 0.263314645 | -0.8782235 |
| ROM | Patch | 1 | 2.2535 | -0.7981564 | -0.93886521 | -0.22605369 |
| ROM | Patch | 2 |  | -2.1012965 | -2.67350679 | -1.10585837 |
| ROM | Patch | 3 |  | 0.1030266 | -1.37493299 | 1.42153631 |
| ROM | Patch | 4 |  | -0.4957024 | -0.39576897 | -0.55330852 |
| ROM | Patch | 5 |  | -1.3101862 | -1.32605804 | -1.13755129 |
| ROM | Patch | 6 |  | 0.2256682 | 0.35772259 | 0.02996904 |
| ROM | Patch | 7 |  | -0.5191882 | -0.27869601 | -0.87139035 |
| ROM | Patch | 8 |  | 0.3571619 | 0.05017207 | 0.70678736 |
| ROM | Patch | 9 |  | 0.531849 | 0.66055859 | 0.21898305 |
| ROM | Patch | 10 |  | -1.0786523 | -1.06719917 | -0.88469872 |
| ROM | Patch | 11 |  | -0.6714257 | -0.59110613 | -0.75706716 |
| ROM | Patch | 12 |  | -0.3308913 | -0.42443152 | -0.16190106 |
| ROM | Patch | 13 |  | -0.41281 | -0.73958957 | 0.0779447 |
| ROM | Patch | 14 |  | -0.8583558 | -0.8840178 | -0.69134066 |
| ROM | Patch | 15 |  | -1.1171005 | -1.05061313 | -1.00310906 |
| ROM | Patch | 16 |  | 0.1695839 | 0.51615823 | -0.2705257 |
| ROM | Patch | 17 |  | -1.1000122 | -1.03273756 | -1.06720198 |
| ROM | Patch | 18 |  | -0.7513826 | -0.39314452 | -0.94279781 |
| ROM | Patch | 19 |  | 0.4137326 | 0.45862396 | 0.31891176 |
| ROM | Patch | 20 |  | -0.2415606 | -0.09126817 | -0.42058359 |

Table S3. Standardized effect size (SES values) of the checkerboard score (C-scores) and the functional dispersion index (FDis) for All traits, Aboveground traits, and Belowground traits at each subplot at the neighborhood scale. Site acronyms: QL = Quebrada El León, LLCHA = Norte Llanos de Challe, CHA = Chañaral de Aceituno, ROM = Romeral.

| Site | Scale | Plot | Subplot | SES values | | | |
| --- | --- | --- | --- | --- | --- | --- | --- |
|  |  |  |  | C-Scores | All traits | Aboveground traits | Belowground traits |
| QL | Neighborhood | 11 | 1 | 3.2093 | -1.050132 | -0.9597628 | -1.22595 |
| QL | Neighborhood | 11 | 2 |  | 1.124355 | 1.1290197 | 1.044495 |
| QL | Neighborhood | 11 | 4 |  | -1.451279 | -1.4110936 | -1.438096 |
| QL | Neighborhood | 18 | 2 | 1.9244 | 0.2858314 | 0.370009 | 0.08405543 |
| QL | Neighborhood | 18 | 4 |  | 0.652897 | 0.6981438 | 0.51622919 |
| QL | Neighborhood | 20 | 1 | 2.2572 | -1.2150777 | -1.2619679 | -0.7544889 |
| QL | Neighborhood | 20 | 2 |  | 0.9384501 | 0.6907807 | 1.2166636 |
| LLCHA | Neighborhood | 11 | 1 | 1.4804 | -0.7414383 | -1.028442 | -0.423223 |
| LLCHA | Neighborhood | 11 | 3 |  | 0.1185127 | 0.381407 | -0.3502239 |
| LLCHA | Neighborhood | 11 | 4 |  | -1.7331078 | -1.553992 | -1.6928733 |
| LLCHA | Neighborhood | 12 | 1 | 1.6779 | 0.5666705 | 0.5121682 | 0.3384017 |
| LLCHA | Neighborhood | 12 | 2 |  | -0.4191314 | -1.2979584 | 0.2291579 |
| LLCHA | Neighborhood | 12 | 4 |  | -0.2297927 | 1.1713698 | -1.0268261 |
| LLCHA | Neighborhood | 13 | 1 | 1.5106 | 1.0278322 | 0.09458668 | 1.6213377 |
| LLCHA | Neighborhood | 13 | 3 |  | -1.3266088 | -1.09550091 | -1.1948565 |
| LLCHA | Neighborhood | 13 | 4 |  | -0.5522406 | -0.65504672 | -0.3432703 |
| LLCHA | Neighborhood | 13 | 5 |  | 1.1347594 | 1.69578407 | -0.938147 |
| LLCHA | Neighborhood | 14 | 1 | 2.6063 | -0.4055898 | -0.5358717 | -0.1719502 |
| LLCHA | Neighborhood | 14 | 2 |  | 0.4093413 | 0.2144289 | 0.6215666 |
| LLCHA | Neighborhood | 14 | 3 |  | 1.6835383 | 2.0434585 | 1.1569769 |
| LLCHA | Neighborhood | 14 | 4 |  | -0.9355615 | -0.8233908 | -1.0382715 |
| LLCHA | Neighborhood | 14 | 5 |  | -0.8104364 | -0.7019892 | -0.9154412 |
| LLCHA | Neighborhood | 15 | 1 | 2.2493 | 0.7753952 | -0.02073406 | 1.2664226 |
| LLCHA | Neighborhood | 15 | 2 |  | 0.1423942 | 0.85335865 | -1.0390497 |
| LLCHA | Neighborhood | 15 | 4 |  | 0.2553328 | 0.09057675 | 0.3855858 |
| LLCHA | Neighborhood | 15 | 5 |  | 0.6400527 | 0.40583913 | 0.697073 |
| LLCHA | Neighborhood | 16 | 1 | 1.5752 | -0.8859928 | -0.9664216 | -0.54022803 |
| LLCHA | Neighborhood | 16 | 2 |  | 0.845654 | 1.1097547 | -0.11771642 |
| LLCHA | Neighborhood | 16 | 3 |  | 1.2061366 | 1.4733673 | 0.51414778 |
| LLCHA | Neighborhood | 16 | 4 |  | -0.7947667 | -1.0955002 | -0.08617606 |
| LLCHA | Neighborhood | 17 | 2 | 0.65991 | -0.940457 | -0.7729041 | -0.9509664 |
| LLCHA | Neighborhood | 17 | 3 |  | 1.772386 | 1.7982527 | 1.7711434 |
| LLCHA | Neighborhood | 19 | 1 | 1.2089 | -2.5509166 | -1.6885211 | -1.7156699 |
| LLCHA | Neighborhood | 19 | 2 |  | -0.4966193 | -1.68134 | 1.5179839 |
| LLCHA | Neighborhood | 19 | 3 |  | 0.6161964 | 0.3004746 | 0.5694886 |
| LLCHA | Neighborhood | 19 | 5 |  | 0.5359893 | 1.3103838 | -1.211558 |
| LLCHA | Neighborhood | 20 | 2 | 1.8823 | -0.7111263 | -0.4579876 | -1.4542268 |
| LLCHA | Neighborhood | 20 | 3 |  | 1.2823602 | -0.1073039 | 1.2690293 |
| LLCHA | Neighborhood | 20 | 4 |  | -0.8294779 | -0.2326612 | -0.6429799 |
| CHA | Neighborhood | 8 | 1 | 1.9521 | 1.08106129 | 1.6461915 | 0.3690549 |
| CHA | Neighborhood | 8 | 2 |  | -1.54082578 | -1.7684968 | -0.446714 |
| CHA | Neighborhood | 8 | 3 |  | -0.65126223 | -0.1282489 | -0.9257591 |
| CHA | Neighborhood | 8 | 4 |  | -0.03618069 | -0.4155202 | 0.3071674 |
| CHA | Neighborhood | 8 | 5 |  | 0.589774 | 0.2782765 | 0.7331279 |
| CHA | Neighborhood | 9 | 2 | 1.1275 | 1.5184385 | 1.3112108 | 1.4643906 |
| CHA | Neighborhood | 9 | 3 |  | -0.9299321 | -1.154801 | -0.6786091 |
| CHA | Neighborhood | 9 | 4 |  | 0.5281245 | 0.436662 | 0.8900105 |
| CHA | Neighborhood | 9 | 5 |  | -0.9134797 | -0.7910685 | -0.9516657 |
| CHA | Neighborhood | 10 | 1 | 1.6916 | -1.7341063 | -1.1603019 | -2.493207 |
| CHA | Neighborhood | 10 | 2 |  | -0.04511461 | -0.5344201 | 1.123598 |
| CHA | Neighborhood | 10 | 3 |  | 1.14729438 | 1.2974109 | 0.578753 |
| CHA | Neighborhood | 10 | 4 |  | -0.40130642 | -0.4070419 | -0.188679 |
| CHA | Neighborhood | 10 | 5 |  | 1.45668892 | 1.30411 | 1.021124 |
| CHA | Neighborhood | 11 | 1 | 1.9233 | 1.4836167 | 1.44943926 | 1.4946689 |
| CHA | Neighborhood | 11 | 3 |  | 0.1204489 | 0.04739033 | 0.1730666 |
| CHA | Neighborhood | 11 | 4 |  | -0.318659 | -0.1626542 | -0.4260775 |
| CHA | Neighborhood | 11 | 5 |  | -1.282199 | -1.34902197 | -1.2401271 |
| CHA | Neighborhood | 12 | 1 | 1.7188 | 0.869775 | 1.1072456 | 0.4172818 |
| CHA | Neighborhood | 12 | 2 |  | 0.3942343 | 0.3382285 | 0.4584144 |
| CHA | Neighborhood | 12 | 3 |  | 1.6006661 | 1.4414888 | 1.4957623 |
| CHA | Neighborhood | 12 | 4 |  | -0.9850776 | -1.1727019 | -0.5319611 |
| CHA | Neighborhood | 12 | 5 |  | -3.4597595 | -3.2138475 | -3.1398942 |
| CHA | Neighborhood | 13 | 1 | 1.5473 | -0.911096 | -0.8408994 | -0.9387523 |
| CHA | Neighborhood | 13 | 2 |  | -1.7489269 | -1.8213483 | -1.6026899 |
| CHA | Neighborhood | 13 | 3 |  | 0.6807885 | 0.4461325 | 0.8491288 |
| CHA | Neighborhood | 13 | 4 |  | 0.1705272 | 0.5176874 | -0.2114503 |
| CHA | Neighborhood | 13 | 5 |  | 0.9366061 | 0.8247818 | 0.9345999 |
| CHA | Neighborhood | 17 | 1 | 1.5148 | 0.5208496 | 0.2100963 | 0.81536713 |
| CHA | Neighborhood | 17 | 2 |  | -0.4367976 | -0.5705851 | -0.04306469 |
| CHA | Neighborhood | 17 | 4 |  | -1.3986302 | -1.6505229 | -0.87932137 |
| CHA | Neighborhood | 17 | 5 |  | -1.7524173 | -1.6165443 | -1.5904362 |
| CHA | Neighborhood | 18 | 1 | 2.3045 | -0.55073648 | -0.38985703 | -0.71454861 |
| CHA | Neighborhood | 18 | 2 |  | -0.02934307 | 0.000233422 | -0.05112497 |
| CHA | Neighborhood | 18 | 3 |  | 1.39129264 | 0.917696879 | 1.76384987 |
| CHA | Neighborhood | 18 | 4 |  | -2.03162154 | -1.961154203 | -1.88124113 |
| CHA | Neighborhood | 18 | 5 |  | -0.38960795 | -0.526245238 | -0.13632938 |
| CHA | Neighborhood | 19 | 1 | 1.6252 | 0.9157127 | 0.1698317 | 1.1306028 |
| CHA | Neighborhood | 19 | 2 |  | 0.5471138 | 0.4130205 | 0.4344835 |
| CHA | Neighborhood | 19 | 3 |  | -0.767418 | -0.2505761 | -1.0345471 |
| CHA | Neighborhood | 19 | 4 |  | -0.766543 | -0.5681054 | -0.8839495 |
| CHA | Neighborhood | 20 | 1 | 2.1152 | -1.2237548 | -1.3037351 | -1.1008765 |
| CHA | Neighborhood | 20 | 2 |  | 1.0637119 | 1.2544468 | 0.8416818 |
| CHA | Neighborhood | 20 | 3 |  | -0.72028 | -0.6135443 | -0.8686808 |
| CHA | Neighborhood | 20 | 4 |  | -0.5160531 | -0.5024982 | -0.4796689 |
| CHA | Neighborhood | 20 | 5 |  | -0.7465703 | -0.9551422 | -0.4538572 |
| ROM | Neighborhood | 1 | 1 | 0.46813 | 0.01992661 | 0.582431 | -0.8571951 |
| ROM | Neighborhood | 1 | 2 |  | 1.26411694 | 0.8236347 | 1.6336174 |
| ROM | Neighborhood | 1 | 3 |  | -2.24107039 | -1.6474195 | -1.6315435 |
| ROM | Neighborhood | 1 | 4 |  | -0.71375858 | -0.2161766 | -1.14551 |
| ROM | Neighborhood | 1 | 5 |  | -1.13815398 | -1.0228699 | -0.812393 |
| ROM | Neighborhood | 2 | 1 | -0.81779 | 1.12875337 | 0.87230068 | 1.4531725 |
| ROM | Neighborhood | 2 | 2 |  | -0.02802277 | 0.03484902 | -0.1833574 |
| ROM | Neighborhood | 2 | 3 |  | -1.51494584 | -1.729751 | -1.0246377 |
| ROM | Neighborhood | 2 | 4 |  | -0.1019918 | -0.0094586 | -0.1985282 |
| ROM | Neighborhood | 2 | 5 |  | 1.03419321 | 1.34297247 | 0.4945907 |
| ROM | Neighborhood | 8 | 1 | 0.17706 | 1.3667298 | 2.799085982 | -2.1618454 |
| ROM | Neighborhood | 8 | 2 |  | -1.6514892 | -1.071856982 | -0.2723468 |
| ROM | Neighborhood | 8 | 3 |  | 0.4253298 | 0.002778153 | 0.807304 |
| ROM | Neighborhood | 8 | 4 |  | 0.496115 | 0.129998506 | 0.7390552 |
| ROM | Neighborhood | 8 | 5 |  | -0.341383 | -1.188181672 | 0.6118071 |
| ROM | Neighborhood | 10 | 1 | 1.4876 | -0.1073172 | 0.2469811 | -0.2377743 |
| ROM | Neighborhood | 10 | 3 |  | -0.4962425 | -0.2332735 | -0.5501103 |
| ROM | Neighborhood | 10 | 4 |  | -0.8678455 | -0.7967449 | -0.7614552 |
| ROM | Neighborhood | 13 | 1 | 2.6726 | 0.1704544 | -0.2192643 | 0.64184032 |
| ROM | Neighborhood | 13 | 2 |  | 1.2949712 | 1.7592671 | -0.07325445 |
| ROM | Neighborhood | 13 | 3 |  | 0.6446117 | -0.7324978 | 1.54368326 |
| ROM | Neighborhood | 13 | 4 |  | -0.365192 | 0.1735306 | -0.73793625 |
| ROM | Neighborhood | 13 | 5 |  | -2.981379 | -2.114902 | -2.04899343 |
